# Dermatitis Herpetiformis: A Common Extraintestinal Manifestation of Coeliac Disease

**DOI:** 10.3390/nu10050602

**Published:** 2018-05-12

**Authors:** Timo Reunala, Teea T. Salmi, Kaisa Hervonen, Katri Kaukinen, Pekka Collin

**Affiliations:** 1 Celiac Disease Research Center, Faculty of Medicine and Life Sciences, University of Tampere, 33014 Tampere, Finland; teea.salmi@staff.uta.fi (T.T.S.); kaisa.hervonen@staff.uta.fi (K.H.); katri.kaukinen@staff.uta.fi (K.K.); 2 Department of Dermatology, Tampere University Hospital, 33521 Tampere, Finland; 3 Department of Internal Medicine, Tampere University Hospital, 33521 Tampere, Finland; 4 Department of Gastroenterology and Alimentary Tract Surgery, Tampere University Hospital, 33521 Tampere, Finland; pekka.collin@uta.fi

**Keywords:** dermatitis herpetiformis, coeliac disease, prevalence, epidermal transglutaminase, gluten-free diet, long-term prognosis

## Abstract

Dermatitis herpetiformis (DH) is a common extraintestinal manifestation of coeliac disease presenting with itchy papules and vesicles on the elbows, knees, and buttocks. Overt gastrointestinal symptoms are rare. Diagnosis of DH is easily confirmed by immunofluorescence biopsy showing pathognomonic granular immunoglobulin A (IgA) deposits in the papillary dermis. A valid hypothesis for the immunopathogenesis of DH is that it starts from latent or manifest coeliac disease in the gut and evolves into an immune complex deposition of high avidity IgA epidermal transglutaminase (TG3) antibodies, together with the TG3 enzyme, in the papillary dermis. The mean age at DH diagnosis has increased significantly in recent decades and presently is 40–50 years. The DH to coeliac disease prevalence ratio is 1:8 in Finland and the United Kingdom (U.K.). The annual DH incidence rate, currently 2.7 per 100,000 in Finland and 0.8 per 100,000 in the U.K., is decreasing, whereas the reverse is true for coeliac disease. The long-term prognosis of DH patients on a gluten-free diet is excellent, with the mortality rate being even lower than for the general population.

## 1. Introduction

Dermatitis herpetiformis (DH) was described as a clinical entity by Louis Duhring in 1884, four years before Samuel Gee published the symptoms of coeliac disease [1,2]. The hallmark of DH is the symmetrical distribution of small vesicles and papules typically on the elbows, knees, and buttocks [3]. An intense itch is common, meaning that patients often scratch the vesicles. A breakthrough in the accurate diagnosis of DH was the discovery of granular immunoglobulin A (IgA) deposits in the skin in 1969 [4]. Though patients with DH rarely presented with overt gastro-intestinal symptoms, small bowel biopsies taken in the 1960s showed villous atrophy identical to that found in coeliac disease [5]. However, a quarter of the patients had normal small bowel villous architecture. Subsequently, it became evident that these patients also had coeliac-type minor enteropathy, i.e., an increased density of gamma/delta intraepithelial lymphocytes [6].

The rash in DH responds to a strict a gluten-free diet (GFD), albeit slowly, and the symptoms recur on gluten challenge [7,8,9]. Therefore, a life-long GFD is the treatment of choice for all patients with DH. Additionally, most patients initially receive dapsone (4,4-diaminodiphenylsulfone) medication, which can be tapered off after a mean of two years’ strict adherence to a GFD [10].

Genetic and family studies tie DH and coeliac disease closely together. Almost every patient with DH and coeliac disease has the alleles contributing to the HLA-DQ2 or HLA-DQ8 haplotype [11]. These diseases segregate in the same families [12] and even monozygotic twin pairs can be affected by DH and coeliac disease [13].

Clinical presentation of DH is not easy to recognize correctly by general practitioners and delay of diagnosis for over two years occur in one third of the Finnish patients [14]. The presence of the blistering rash with IgA deposits is the major difference between DH and coeliac disease. However, other differences exist such as gender, age at onset, incidence, and long-term prognosis on a GFD. These points will be discussed in more detail along with the immunopathogenesis of DH.

## 2. Clinical Presentation and Diagnosis of Dermatitis Herpetiformis

**Figure 1:**
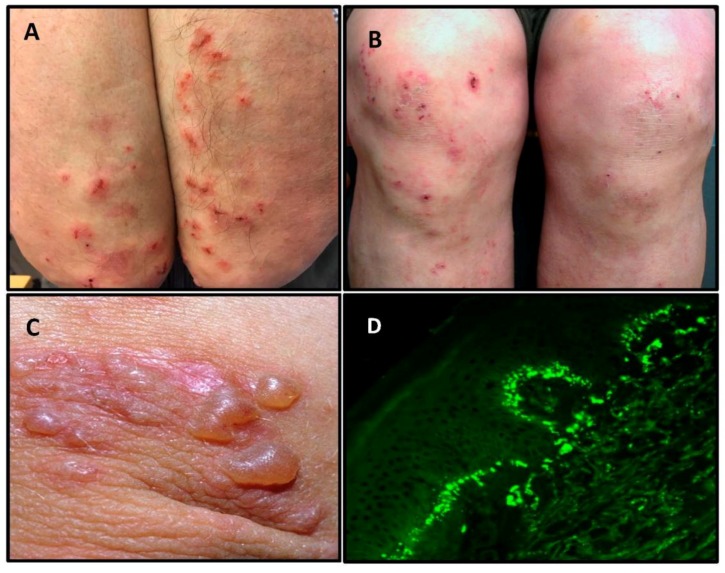
Dermatitis herpetiformis. Typical scratched papules and macules on the elbows (**A**), and on the knees (**B**). Fresh small blisters on the elbow (**C**). Direct immunofluorescence showing granular IgA deposits in the basal membrane zone between epidermis and dermis (**D**).

The typical sites of predilection of DH are the extensor surfaces of elbows and knees, and the buttocks (Figure 1A,B). In addition, the upper back, abdomen, scalp and face can be affected, but oral lesions are rare [3]. The rash is polymorphic with small blisters (Figure 1C). These are, however, often eroded and crusted because of intense itch and scratching. Purpuric lesions may also appear on the hands and feet, however, this is rare [3]. The presentation and activity of the rash varies greatly from patient to patient, but complete remission is infrequent on a normal, gluten-containing diet.

The clinical picture is often highly suggestive of DH, although, linear IgA bullous disease is always a diagnostic problem [15]. Itchy skin disorders such as urticaria, atopic or nummular dermatitis, and scabies infestation should be considered as a differential diagnosis [3]. The localization and burning itch experienced during the development of blisters is, however, usually severe enough to raise suspicion of DH. The typical histopathological findings in the lesional skin of patients with DH consists of subepidermal vesicles associated with an accumulation of neutrophils at the papillary tips. The histopathology of a DH skin lesion is not diagnostic since other bullous diseases, such as linear IgA bullous disease and epidermolysis bullosa acquisita, may show similar findings [16]. Moreover, the histopathologic picture of DH is often unspecific revealing only perivascular lymphocytic infiltrate and minimal inflammation in dermal papillae.

The ideal method for diagnosis of DH is a direct immunofluorescence biopsy of unaffected skin in close proximity to an active lesion [17]. This reveals pathognomonic granular IgA deposits at the dermo-epidermal junction (Figure 1D), and the diagnosis of DH should not be made without this finding [16].

## 3. Gender and Age at Onset

Earlier studies in adults with DH have shown male to female ratios ranging up to 2:1 [3] with two recent large DH studies finding the ratio to be close to 1:1 [18,19]. This is in sharp contrast to coeliac disease, in which females outnumber males [20] (Table 1). This gender imbalance may reduce with increasing age, and it has also been absent in some coeliac disease screening studies [21,22]. These findings suggest that gender differences between DH and coeliac disease are perhaps not as profound as was earlier thought.

Coeliac disease can be diagnosed at any age with the peak incidences being in early childhood and between 40 and 60 years of age [20,23]. Clinical series in adults with DH from Europe and North America have shown that the mean age at diagnosis is between 40 and 50 years [14,24]. Like coeliac disease, the oldest DH patients have been over 80 years of age at diagnosis. In contrast to coeliac disease, DH in childhood seems to be rare; it was found in only 4% of 476 Finnish patients [25]. However, differences may exist. In an Italian series comprising 159 DH patients, 36% were below the age of 20 years [26], and a large series of 127 Hungarian children with DH has been published [27].

A study of 477 patients collected from 1970 onwards in Finland showed a significant increase in the mean age at diagnosis [18]. The increase was from 35 to 51 years in men and from 36 to 46 years in women. A similar increasing trend in the mean age at diagnosis has also been observed in recent decades in adult coeliac disease, both in Finland and elsewhere [28,29,30]. One explanation for this trend may be changes in dietary habits, such as the consumption of wheat, which in Sweden has changed the appearance of childhood coeliac disease [31]. In Finland, the annual consumption of wheat, rye, and other cereals per person has decreased from 150 kg to 71 kg over the past 50 years [32]. A lower lifetime gluten load might thus explain the increasing age at diagnosis and perhaps also the trend towards less severe small bowel atrophy in DH and coeliac disease [33,34,35].

## 4. Prevalence and Incidence

Two recent large DH studies with a total of 477 and 809 patients found a prevalence of 75.3 per 100,000 in Finland and 30 per 100,000 in the U.K., respectively [18,19] (Table 1). In the U.K. study the prevalence of coeliac disease was 240 per 100,000, i.e., eight times higher than that of DH. The same 8:1 ratio was calculated in the Finnish study, where the national prevalence of coeliac disease was 661 per 100,000 [38]. Nevertheless, DH is evidently the most common extraintestinal manifestation of coeliac disease [42].

**Figure 2:**
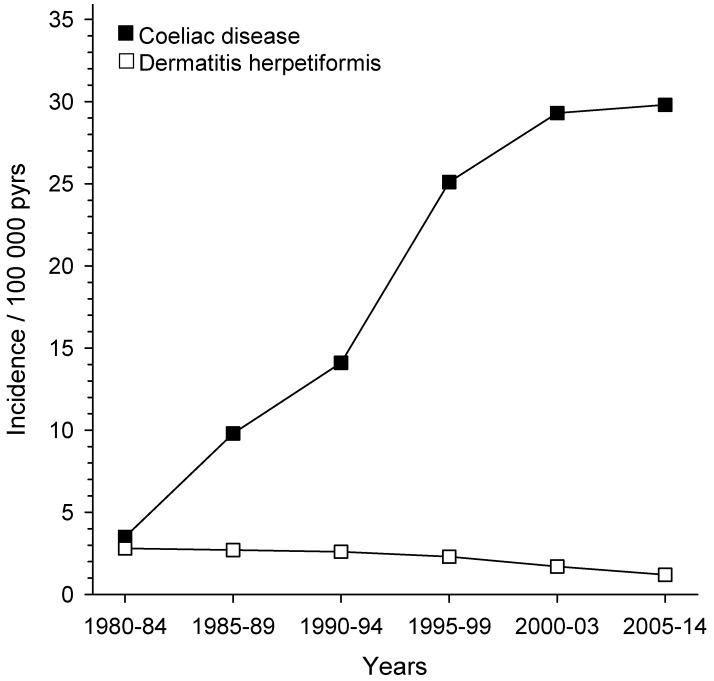
Incidence of dermatitis herpetiformis and coeliac disease in Finland, 1980–2014. The data include 3671 adult patients with Dermatitis herpetiformis and 31,385 adult patients with coeliac disease registered with the Social Insurance Institution of Finland [28,43].

The Social Insurance Institution of Finland maintains a nationwide register of adults with coeliac disease and DH. In 2003, it included a total of 18,538 patients, of whom 3121 (17%) had DH [28]. When we compared the annual numbers of new cases in five-year periods from 1980 to 2003, it was evident that in the first period patients with coeliac disease only slightly outnumbered those with DH (Figure 2). After the first period, the annual number of DH patients slowly decreased, whereas the number of coeliac disease patients continuously increased. In the ten-year period of 2005–2014, the proportion of newly diagnosed patients with DH had decreased to only 4% [43] (Figure 2).

Importantly, a Finnish cohort study [18] and a U.K. register study [19] covering time periods of 30 and 20 years, respectively, showed convincingly that the annual incidence rate of DH has decreased significantly. This decrease was from 5.2 to 2.7 per 100,000 in the Finnish study and from 1.8 to 0.8 per 100,000 in the U.K. study. The opposite was true for coeliac disease; there was a maximum of a fourfold increase in the incidence rate [19]. The reason for this seems to be an increasing awareness of mild symptoms, the development of efficient serological screening tests, and the identification of special risk groups, such as family members [20,23]. Sero-epidemiological studies suggest that in addition to the better recognition of coeliac disease, there has also been a true increase in the incidence in recent decades [44,45].

The decreasing incidence rate of DH in Finland and the U.K., along with a simultaneous rapid increase in coeliac disease, fits our hypothesis that subclinical, undiagnosed coeliac disease is a prerequisite for the development of DH. In support of this hypothesis, we know of patients initially diagnosed with coeliac disease who did not follow or only partially followed a GFD and subsequently developed DH [46]. Moreover, adult patients with DH frequently have coeliac-type dental enamel defects, which develop early in childhood as a result of malabsorption or immune alteration caused by undiagnosed coeliac disease [47].

## 5. Pathogenesis of Dermatitis Herpetiformis: From Gut to Skin

In DH, pathognomonic granular IgA deposits in the papillary dermis have long been suspected to derive from the gut. In 2002, Sárdy et al. [48] showed that the autoantigen for deposited cutaneous IgA is epidermal transglutaminase (TG3). This is closely related, but not identical, to the tissue transglutaminase (TG2) autoantigen specific for coeliac disease [49]. The TG2 enzyme is a target for IgA class autoantibody deposition in the small bowel mucosa in classical and potential coeliac disease, and in DH [36,37,50]. The TG3 protein has not been detected in the small bowel similarly to the TG2 enzyme, but epitope spreading is a possibility [51]. Recently, DH patients with the active disease have been shown to secrete high levels of TG3 antibodies into the gut organ culture medium, and also have TG3-antibody-positive cells in the small intestinal mucosa [52]. At present, a valid hypothesis is that the immunopathogenesis of DH starts from hidden coeliac disease in the gut with a TG2, and possibly also a TG3, autoantibody response and evolves into an immune complex deposition of high avidity IgA TG3 antibodies together with the TG3 enzyme in the papillary dermis [48]. Further support for this comes from GFD treatment results; TG3 and TG2 antibodies in the blood disappear with the dietary treatment and, at the same time, the rash and small bowel heal [53]. In contrast, the IgA-TG3 aggregates in the skin disappear very slowly with GFD treatment [54]. This seems to be due to the active TG3 enzyme in the aggregates resulting in covalent cross-linking of the complex to dermal structures [55].

## 6. Long-Term Prognosis on a Gluten-Free Diet Treatment

A GFD is the treatment of choice for all patients with DH regardless of whether they have villous atrophy in the small bowel [7,8,10]. It is important to know the long-term prognosis for DH because, as in coeliac disease, the risk of non-Hodgkin’s lymphoma is significantly increased [56]. However, a strict GFD for more than five years seems to protect against lymphoma [57]. In agreement with this, our cohort of 476 patients with DH, where almost all patients adhered to a GFD, showed a significantly increased lymphoma mortality rate during the first five years of follow-up, but not thereafter [39]. Unexpectedly, the same study showed that the standardized mortality rate (SMR 0.70) was statistically significantly reduced in the GFD-treated DH patients compared to the general population. In a previous study of 846 DH patients from the U.K. [40], the adherence to a GFD was only partly known, and the mortality rate was slightly, but non-significantly, reduced (hazard ratio 0.93).

In contrast to the excellent long-term prognosis for DH, a meta-analysis of prospective studies on coeliac disease found a significantly increased risk for all-cause (odds ratio 1.24) and non-Hodgkin’s lymphoma (odds ratio 2.61) mortality [41]. A recent large register study from England could only confirm the excess risk of deaths from non-Hodgkin’s lymphoma [58]. However, in coeliac disease mortality studies, the adherence to a GFD has not been analyzed or was only partially known [41,59]; therefore, there is a particular need to examine the relationship between mortality rate and dietary compliance.

There are studies suggesting that patients with coeliac disease on a GFD retain a reduced quality of life compared to the general population [60,61]. Recently, we examined long-term GFD-treated patients with DH [62]. Their quality of life was comparable to that of the general population, whereas it was reduced in the coeliac disease controls.

## 7. Concluding Remarks

DH is the most common extraintestinal clinical manifestation of coeliac disease; at present, the prevalence ratio of the disorders is 1:8. The incidence of DH is decreasing, whereas the opposite is true for coeliac disease. A valid current hypothesis is that subclinical coeliac disease is a prerequisite for the development of DH. The reason why only some undiagnosed coeliac individuals develop an itchy blistering rash with dermal IgA-TG3 deposits remains unknown. The markedly increased age at diagnosis and less severe small bowel damage both in DH and coeliac disease suggests changes in environmental factors, such as a lowered lifetime gluten load. The long-term prognosis of DH patients on a GFD is excellent, which seems to be due to a strict adherence to the diet.

## Figures and Tables

**Table 1:** Differences between dermatitis derpetiformis and coeliac disease.

	Dermatitis Herpetiformis	Coeliac Disease
Gender	Slightly more males	Females predominate
Age at onset	Mainly adults	Children and adults
IgA-TG3 deposits in the skin	100%	0%
Small bowel villous atrophy	75%	100% *
IgA-TG2 deposits in the small bowel mucosa [36,37]	80%	up to 100% **
Prevalence in Finland and United Kingdom [18,19,38]	75 and 30 per 100,000	660 and 240 per 100,000
Incidence	Decreasing	Increasing
Response to a gluten-free diet [7,8,20]	Slow; months, in the beginning most patients need dapsone to control the rash	Rapid; days or weeks until gastro-intestinal symptoms end whereas small bowel villous atrophy may persist for many years
Long-term prognosis on a gluten-free diet [39,40,41]	Excellent	All-cause and lymphoma mortality may be increased

TG3 = epidermal transglutaminase, TG2 = tissue transglutaminase; * Potential coeliac disease with normal small bowel mucosa architecture, inflammation and positive TG2 serology also exist; ** Data still sparse.
